# Nanoemulsion‐Loaded Capsules for Controlled Delivery of Lipophilic Active Ingredients

**DOI:** 10.1002/advs.202001677

**Published:** 2020-08-28

**Authors:** Liang‐Hsun Chen, Li‐Chiun Cheng, Patrick S. Doyle

**Affiliations:** ^1^ Department of Chemical Engineering Massachusetts Institute of Technology 77 Massachusetts Avenue Cambridge MA 02139 USA; ^2^ Campus for Research Excellence and Technological Enterprise Singapore 138602 Singapore

**Keywords:** capsules, controlled release, core–shell hydrogels, liphophilic active ingredients, nanoemulsions

## Abstract

Nanoemulsions have become ideal candidates for loading hydrophobic active ingredients and enhancing their bioavailability in the pharmaceutical, food, and cosmetic industries. However, the lack of versatile carrier platforms for nanoemulsions hinders advanced control over their release behavior. In this work, a method is developed to encapsulate nanoemulsions in alginate capsules for the controlled delivery of lipophilic active ingredients. Functional nanoemulsions loaded with active ingredients and calcium ions are first prepared, followed by encapsulation inside alginate shells. The intrinsically high viscosity of the nanoemulsions ensures the formation of spherical capsules and high encapsulation efficiency during the synthesis. Moreover, a facile approach is developed to measure the nanoemulsion release profile from capsules through UV–vis measurement without an additional extraction step. A quantitative analysis of the release profiles shows that the capsule systems possess a tunable, delayed‐burst release. The encapsulation methodology is generalized to other active ingredients, oil phases, nanodroplet sizes, and chemically crosslinked inner hydrogel cores. Overall, the capsule systems provide promising platforms for various functional nanoemulsion formulations.

## Graphical abstract

A method is developed to encapsulate nanoemulsions in alginate capsules for the controlled delivery of lipophilic active ingredients. The nanoemulsions are intrinsically viscous, ensuring the formation of spherical capsules and large encapsulation efficiency. The capsule systems possess a tunable, delayed‐burst release. The methodology is generalized to other active ingredients, oil phases, nanodroplet sizes, and chemically crosslinked inner hydrogel cores.

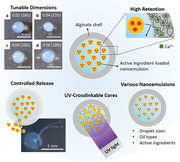

## 1 Introduction

Nanoemulsions have gained considerable attention in recent years because of the increasing need to develop effective delivery systems for lipophilic active ingredients. These lipophilic active ingredients are ubiquitous in a wide variety of applications, such as pharmaceutical manufacturing,^[^ ^1^, ^2^, ^3^ ^]^ cosmetic formulations,^[^ ^4^ ^]^ and food processing.^[^ ^5^, ^6^ ^]^ In pharmaceutical applications, it is known that 40% of currently marketed drugs and 90% of drugs in development are hydrophobic^[^ ^7^ ^]^ wherein the low water solubility greatly limits their bioavailability and absorption efficiency. Oil‐in‐water (O/W) nanoemulsion systems have been extensively pursued for hydrophobic pharmaceutical compounds. These nanoemulsions are dispersed oil droplets with an average size ranging from 20 to 500 nm in an aqueous continuous phase.^[^ ^8^ ^]^ The oil nanodroplets can act as effective reservoirs for solubilizing various lipophilic ingredients and protecting them from degradation due to external factors, such as oxidation, pH, or hydrolysis.^[^ ^9^ ^]^ In contrast to conventional macroemulsions, a nanoemulsion has uniformly small nanodroplets that are more resistant to flocculation and coalescence.^[^ ^5^ ^]^ Moreover, their improved kinetic stability enables a nanoemulsion to possess a longer shelf life, and their larger active surface area to volume of droplets enables better availability of incorporated active ingredients. For oral drug delivery, the high loading capacity of an O/W nanoemulsion to solubilize a hydrophobic drug has shown a significant improvement of the bioavailability compared to the unformulated crystalline form.^[^ ^10^ ^]^ O/W naonemulsions are used to enhance lipid digestibility and the absorption of bioactive ingredients in food products and supplements.^[^ ^11^ ^]^ The efficacy of cosmetic products can also be greatly improved with oil nanodroplets leading to enhanced permeation of active ingredients into skin.^[^ ^12^ ^]^

Advances in encapsulation technologies have facilitated the formulation of active ingredients into versatile dosage forms, in which the active ingredients can be carried for accurate administration,^[^ ^13^ ^]^ controlled delivery,^[^ ^14^, ^15^, ^16^, ^17^ ^]^ and enhanced stability.^[^ ^18^ ^]^ Hydrogel encapsulation is one of the most promising routes and has been widely applied to design delivery platforms for various administration routes, including oral, parenteral, and topical routes.^[^ ^19^ ^]^ Hydrogels are 3D polymeric networks that can be readily customized and formulated into particles with various sizes and shapes.^[^ ^19^ ^]^ Their tunable physical properties facilitate the development of novel drug delivery platforms.^[^ ^20^ ^]^ Alginate hydrogels are natural polysaccharides that have gained increasing interest because of their nontoxicity and biocompatibility.^[^ ^21^ ^]^ In addition, the ionic crosslinking of alginate via divalent cations is facile and gentle on incorporated actives. Thus, alginate hydrogels are widely applied in food, pharmaceutical, and biomedical industries.^[^ ^22^ ^]^ However, the hydrophilic nature of hydrogels inherently limits their ability to encapsulate hydrophobic active ingredients. To overcome this limitation, major research efforts have been focused on incorporating hydrophobic domains into hydrogel matrices by encapsulating nanoparticles,^[^ ^23^ ^]^ macroemulsions,^[^ ^24^ ^]^ and nanoemulsions into hydrogel particles. For example, alginate beads have been used to encapsulate macroemulsions for controlled lipid digestion.^[^ ^25^, ^26^ ^]^ The encapsulation efficiency of lipid droplets depends on the degree of alginate crosslinking.^[^ ^27^ ^]^ Recently, our group encapsulated nanoemulsions inside alginate beads for confined crystallization.^[^ ^3^, ^28^, ^29^, ^30^ ^]^ After solvent evaporation, nanocrystals of a lipophilic active pharmaceutical ingredient (API) were formed with the crystal size dictated by the oil droplet size. In addition, nanoemulsion‐loaded alginate beads have also been applied for delivering a lipophilic bioactive ingredient in the liquid form.^[^ ^31^ ^]^ However, the encapsulation of nanoemulsions in alginate beads becomes more challenging as the droplet sizes are reduced to nanoscale.^[^ ^31^ ^]^ A large number of oil nanodroplets, especially when their sizes are close to the alginate mesh size (≈10 nm),^[^ ^26^ ^]^ could potentially hinder the alginate crosslinking and disrupt the hydrogel structure, leading to significant leakage of the nanoemulsion.

To the best of our knowledge, nanoemulsion encapsulation techniques are limited to monolithic dosage forms.^[^ ^3^, ^28^, ^29^, ^30^, ^31^, ^32^ ^]^ Here, we report a new method to encapsulate nanoemulsions in alginate capsules for the controlled delivery of lipophilic active ingredients. After the addition of CaCl_2_ into nanoemulsions, a simple one‐batch inverse gelation technique^[^ ^33^ ^]^ is applied by dripping the calcium‐laden nanoemulsions into an alginate bath. The nanoemulsion suspensions prepared in our work have an intrinsically large viscosity which enables their encapsulation in alginate capsules. In addition, a facile method is proposed to measure the release of nanoemulsions which avoids a tedious extraction step and facilitates automated real‐time measurements using a United States Pharmacopeia (USP) dissolution apparatus. We demonstrated that alginate capsules have improved encapsulation efficiency compared to their monolithic bead counterparts. The bursting time of the alginate capsules is related to the shell properties, which can be easily engineered by varying the calcium concentration during synthesis. The capsule systems are also generalized to other combinations of active ingredients and oils, and tailored nanodroplet sizes. With the addition of UV‐crosslinkable precursors, the inner nanoemulsion liquid cores can be chemically crosslinked into a nanoemulsion‐laden hydrogel for the preparation of core–shell hydrogel particles. The proposed capsule systems provide an advanced platform for the encapsulation and controlled release of nanoemulsions.

### 1.1 Preparation of Calcium API‐Loaded Nanoemulsions and Their Encapsulation in Alginate Capsules

**Figure 1:**
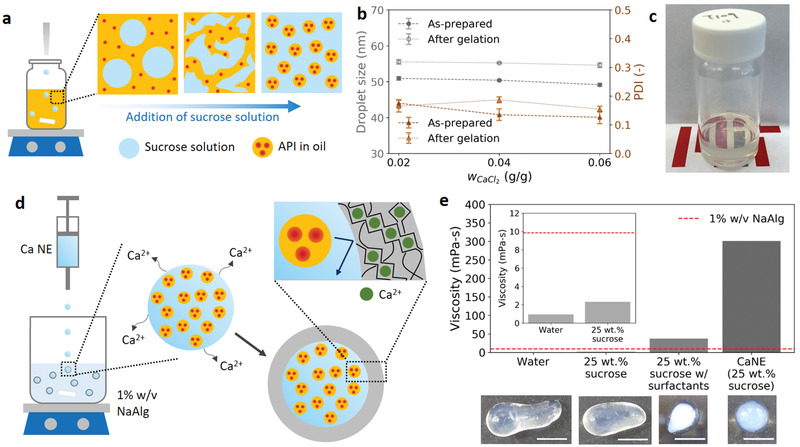
Overview of the nanoemulsion and capsule synthesis. a) Schematic diagram of the low‐energy nanoemulsion preparation. Ibuprofen‐loaded isopropyl myristate (oil phase) added with surfactants are slowly diluted with 25 wt% sucrose solution. b) Droplet size and polydispersity index for the as‐prepared and postgelation calcium‐added nanoemulsions containing calcium concentrations $w_{\mathrm{CaC}l_{2}}$of 0.02, 0.04, and 0.06 g g^−1^ nanoemulsion. c) Optical image of an as‐prepared calcium‐added nanoemulsion in a glass vial. d) Schematic diagram of the formation of alginate capsules for nanoemulsion encapsulation. The calcium ions diffuse out and ionically crosslink the alginate polymers. e) Viscosity of the nanoemulsion solution used to produce spherical capsules compared to the viscosity of water, 25 wt% sucrose, and the nanoemulsion continuous phase, each containing 0.2 g of CaCl_2_. The dotted red line represents the viscosity of the 1% w/v alginate bath. Corresponding images for the capsules prepared with each solution are shown here. The scale bar is 5 mm.

To encapsulate a nanoemulsion containing an API in an alginate capsule, a nonionic nanoemulsion loaded with an API is first prepared using a low‐energy phase inversion method,^[^ ^34^ ^]^ followed by the addition of CaCl_2_ into the aqueous continuous phase. Ibuprofen is used as a model API because of its poor water solubility (0.021 mg mL^−1^ at 25 *°*C)^[^ ^35^ ^]^ and is dissolved in isopropyl myristate (100 mg mL^−1^) as the oil phase. The water phase containing 25 wt% sucrose solution is slowly dripped into a mixture of the oil phase and surfactants (a mixture of 81.3 wt% Tween 80 and 18.7 wt% Span 80 with a hydrophilic‐lipophilic balance (HLB) value of 13) (**Figure** **1**a). The resulting nanoemulsion has an oil weight fraction of ≈20 wt% and a surfactant‐to‐oil ratio (SOR) of 1. During the dilution process, the system passes through an inversion point where the interfacial tension of the oil–water interface is significantly decreased, and thus small droplets are formed. Sucrose is chosen as a small molecule thickener to slightly increase the nanoemulsion viscosity for the subsequent formation of spherical capsules. Viscosifying the continuous phase has been reported to decrease the nanodroplet size which leads to enhanced long‐term nanoemulsion stability by mitigating gravitational separation and droplet coalescence.^[^ ^11^ ^]^ To enable the capsule gelation process, calcium chloride is added into the nanoemulsion. In this study, nanoemulsion dispersions containing various calcium concentrations are prepared. Because the oil nanodroplets are stabilized by nonionic surfactants, the introduction of calcium ions into the continuous aqueous phase does not perturb the system. The droplet size for each as‐prepared calcium containing nanoemulsion is ≈50 nm (Figure 1b). The polydispersity index (PDI) of each calcium nanoemulsion varies between 0.1 and 0.2, which lies in the typical range for nanoemulsion systems.^[^ ^8^ ^]^ Figure 1c shows an optical image of an as‐prepared calcium nanoemulsion in a glass vial. The nanoemulsion is optically transparent because the small droplets remain colloidally stable and weakly scatter visible light. Capsules are synthesized by dripping the calcium nanoemulsion into an alginate bath (Figure 1d). When a droplet containing nanoemulsion and calcium ions enters the bath, the calcium ions quickly diffuse out and crosslink the alginate polymers around the droplet into a thin shell. The alginate shell prepared from 1% w/v sodium alginate solution has a mesh size of about 10 nm^[^ ^26^ ^]^ and is able to entrap nanodroplets. Excess calcium ions continue diffusing out, and the alginate shell grows until the calcium source supplied in the droplet is depleted. During the gelation process, the alginate bath remains clear with no observable signal of the active ingredient detected by the UV–vis spectrometer. After the gelation, the nanoemulsion inside the capsules is pipetted out for dynamic light scattering (DLS) measurements to determine the droplet size. The nanoemulsion droplet size is observed to increase slightly with a mean droplet size of 55 nm and a PDI still ranging between 0.1 and 0.2 (Figure 1b). It is noted that the nanoemulsion still remains stable despite the composition change in the continuous phase. This exceptional stability can be attributed to the kinetic stability of nanoemulsions, which enables them to be less sensitive to environmental changes compared to microemulsions.^[^ ^36^ ^]^

Although alginate capsules have been extensively applied to encapsulate different materials, forming spherical capsules via droplet dripping still remains difficult because of the high viscosity of an alginate solution which deforms impinging droplets into nonspherical shapes. Therefore, the calcium precursor solutions are generally viscosified with a nongelling polymer.^[^ ^37^, ^38^ ^]^ The viscosity of an emulsion suspension diverges asymptotically with increasing oil fraction. The droplets become closely packed and their motions are severely constrained.^[^ ^39^, ^40^ ^]^ Compared to a macroemulsion, a nanoemulsion has a much higher effective volume fraction than its nominal oil volume fraction. The surfactant shell (with the thickness of *δ*) around the oil core (with the radius of *a*) can greatly enhance the nominal oil volume fraction by a factor of (1 + *δ*/*a*)^3^.^[^ ^41^ ^]^ This unique rheological property renders a concentrated nanoemulsion intrinsically highly viscous, and thus only a minor modulation of the continuous phase viscosity is required to produce spherical capsules. In our work, the as‐prepared nanoemulsions have a droplet diameter of 2(*δ* + *a*) ≈ 50 nm, and the *δ* for Tween 80 (the main and larger surfactant in the mixture) is ≈3 nm.^[^ ^42^ ^]^ These dimensions render the “effective oil fraction” to be about 1.50‐fold higher than the nominal oil fraction. To demonstrate the rheological benefit of the nanoemulsions in the capsule gelation process, we compare the canonical nanoemulsion formulation to three calcium containing precursor solutions prepared from pure water, a 25 wt% sucrose solution, and a 25 wt% sucrose solution containing the same surfactants at the concentration used in the nanoemulsions. The viscosity–shear rate flow curves for these fluids are shown in Figure S1 (Supporting Information). For alginate gelation, when a liquid droplet penetrates into the gelation bath, it can create a cavity on the bath surface in about 10 ms.^[^ ^43^ ^]^ With the cavity depth having the same length scale as the droplet diameter,^[^ ^43^ ^]^ the liquid droplet experiences a shear rate on the order of 100 s^−1^. Therefore, the reported viscosity values are averaged across shear rates between 10 and 1000 s^−1^. Figure 1e shows the viscosity for each calcium precursor solution and the corresponding capsule prepared through the dripping process shown in Figure 1d. It is noted that the addition of sucrose in water can only increase the viscosity from 0.95 to 2.32 mPa s, which is still much smaller than the viscosity of the 1% w/v alginate bath (9.85 mPa s). Therefore, the capsules are severely elongated for these two solutions. For the 3 g sucrose solution (25 wt%) with added 1 g surfactants (a mixture of 81.3 wt% Tween 80 and 18.7 wt% Span 80 with an HLB value of 13), the viscosity is about four times higher than that of the alginate bath, and more spherical capsules can be prepared. However, a large proportion of the capsules still have a distorted pear‐like appearance. In contrast, the nanoemulsion dispersion has a viscosity of 301 mPa s which ensures the formation of uniformly spherical capsules (more discussion is in Section S1 in the Supporting Information).

### 1.2 Size and Shape Analyses of Capsules Prepared under Different Conditions

**Figure 2:**
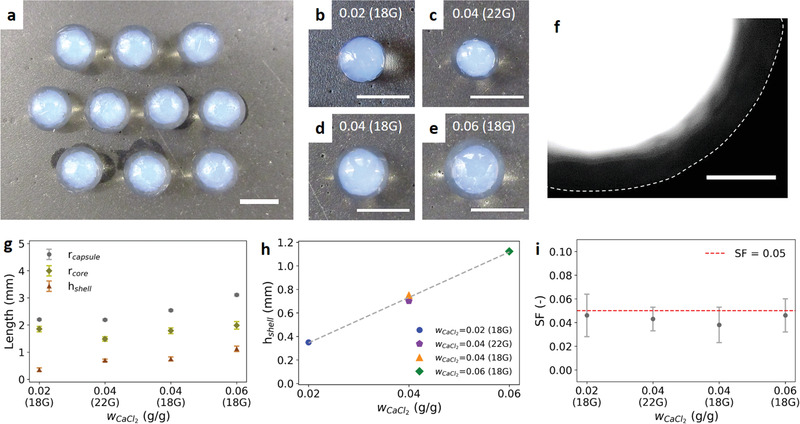
Overview of the nanoemulsion‐laden capsule dimensions. Weight fraction of calcium chloride $w_{\mathrm{CaC}l_{2}}$ is defined as the calcium chloride mass divided by 5 g of naonemulsion. a) Optical images of capsules prepared with $w_{\mathrm{CaC}l_{2}}$= 0.06 and an 18 gauge (18G) dispensing tip. b–e) Zoomed‐in optical images of capsules prepared using different preparation conditions: b) $w_{\mathrm{CaC}l_{2}}$= 0.02 and 18 gauge dispensing tip, c) $w_{\mathrm{CaC}l_{2}}$= 0.04 and 22 gauge dispensing tip, d) $w_{\mathrm{CaC}l_{2}}$= 0.04 and 18 gauge dispensing tip, and e) $w_{\mathrm{CaC}l_{2}}$= 0.06 and 18 gauge dispensing tip. f) Fluorescence microscopy image of a capsule prepared with a lipophilic dye added into the oil phase to label nanodroplets. The white dotted line is the capsule boundary determined from the bright field microscopy image. g) Capsule dimensions (outer radius, core radius, and shell thickness) of capsules prepared under different conditions. h) Linear correlation between shell thickness and $w_{\mathrm{CaC}l_{2}}$. i) Sphericity factors (SF) of the capsules for different preparation conditions. Scale bars for panels (a)–(e) and panel (f) are 5 and 1 mm, respectively.

With an ideally high viscosity, the nanoemulsion dispersion can be easily encapsulated into uniformly spherical alginate capsules (**Figure** **2**a). The transparent ring and blue‐shining inner regions indicate the alginate shell and the inner API‐loaded nanoemulsion, respectively. The nanoemulsion turns blue when the images are taken against a black background (Figure S2, Supporting Information). This phenomenon can be attributed to Rayleigh scattering, which happens when the capsules are observed away from the light source and the nanodroplets are much smaller than the wavelength of visible incident light.^[^ ^44^ ^]^ Blue light of a shorter wavelength is scattered more strongly than other light of longer wavelengths. Figure 2b–e shows the capsules prepared under the four different conditions (capsules in groups are shown in Figure S3 (Supporting Information)). With the same 18 gauge (18G) dispensing tip (Figure 2b,d,e), the inner cores look similar in size but the shell thickness increases with increasing $w_{\mathrm{CaC}l_{2}}$. The inner core size can also be tuned by controlling the size of the dispensing tip (Figure 2c,d). As mentioned before, the alginate mesh size is smaller than the oil nanodroplets. In addition to the optical images that clearly show the two different regions for the inner core and outer shell, a lipophilic dye is also introduced into the oil phase to label the nanodroplets in a capsule. Figure 2f shows the fluorescence microscopy image of a capsule with dyed nanodroplets. The inner core shows a strong fluorescence while the outer shell still remains black, which again demonstrates the effective encapsulation of nanoemulsions in the capsules with our technique. Figure 2g further shows the capsule radius, shell thickness, and core radius that are determined using ImageJ image analysis software. The quantitative results match our observation that the core radii are very similar for the same 18G dispensing tip for the different calcium concentrations; however, the shell thickness is proportional to the $w_{\mathrm{CaC}l_{2}}$ (Figure 2h). Previous work also finds a similar trend and explained that a higher calcium concentration across the shell leads to a larger gradient for calcium ions to diffuse out, and thus a thicker shell can be formed.^[^ ^38^ ^]^ Holding $w_{\mathrm{CaC}l_{2}}$constant at a value of 0.04, dispensing tips with different sizes are utilized to control inner core radii. The relationship between the tip size and inner core size can be described by Tate's law,^[^ ^45^ ^]^ in which the inner core size is proportional to the cube root of the tip size (Figure S5, Supporting Information). Sphericity factors are also determined from the maximum and minimum Feret diameters of the capsules. The sphericity factors for all the conditions are lower than a threshold value of 0.05 (Figure 2i), below which the capsules are considered spherical.^[^ ^45^ ^]^

### 1.3 Development of Release Tests and Controlled Release for Different Preparation Conditions

**Figure 3:**
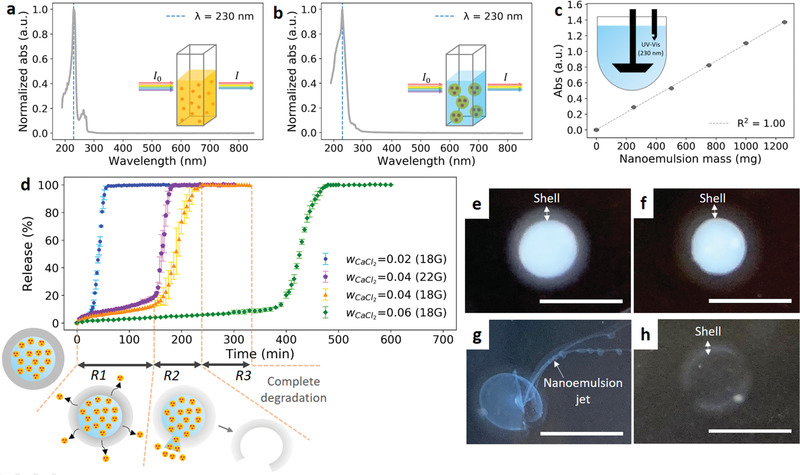
Overview of nanoemulsion release experiments. UV–vis spectra of a) bulk oil phase (100 mg ibuprofen mL^−1^ isopropyl myristate) and b) nanoemulsion prepared from the bulk oil phase. c) Calibration curve for absorbance and nanoemulsion mass at *λ* = 230 nm measured by the UV–vis spectrometer of the dissolution apparatus. The fitted linear curve has a slope of 1.092  × 10^–3^. d) Release profiles using a USP Dissolution Apparatus II of capsules for different preparation conditions. The schematic images below the release profiles depict the release mechanisms for different regimes corresponding to the $w_{\mathrm{CaC}l_{2}}$= 0.04 and 18 gauge curve (which also apply to other curves). Regime *R*1 is diffusion through the shell. Regime *R*2 is the onset of bursting. Regime *R*3 is the postrelease. e–h) Optical images of capsules ($w_{\mathrm{CaC}l_{2}}$= 0.04 and 18G dispensing tip) in a 37 °C saline bath at different times after being added: e) *t* = 0 min, f) in the late *R*1 regime (*t* ≈140 min), g) in *R*2 regime (*t* ≈ 200 min) when the shell has burst, and h) in *R*2 regime (*t* ≈ 200 min) when the nanoemulsion is depleted. Scale bars are 5 mm.

Controlled release is a potential benefit provided by encapsulating a nanoemulsion in alginate capsules. A common and straightforward method utilized to monitor release in prior studies is to combine solvent extraction and UV–vis measurement.^[^ ^31^ ^]^ However, with this method it is difficult to measure the release in real time in an automated manner. To develop an alternative method, we first compare the UV–vis spectra of the bulk API‐loaded oil phase and the nanoemulsion prepared from only the oil phase (**Figure** **3**a,b). Both samples have a characteristic peak occurring at 230 nm. In the nanoemulsion, the Tween 80 also shows strong absorption at this wavelength while the continuous phase materials (sucrose and alginate) have negligible signals (Figure S6, Supporting Information). The UV–vis results indicate that we could possibly apply UV–vis spectroscopy to directly measure the release of the nanoemulsion droplets (and micelles) that consist of the surfactants and dispersed phase. To demonstrate the applicability of this idea, a calibration curve is developed between the UV–vis absorbance and the nanoemulsion mass added into the dissolution apparatus (Figure 3c). A nanoemulsion dispersion with a known mass is added sequentially into the release vessel filled with a 900 mL saline solution, and the corresponding absorbance is recorded. A linear trend is observed for the calibration curve. In our release experiments, the nanoemulsions are substantially diluted in the release vessel and the resulting oil volume fraction is ≈10^−4^ (v/v), which can minimize the light‐scattering events resulting from the nanoemulsions.^[^ ^44^ ^]^ With the linear calibration curve, release tests are conducted to obtain the release profiles of capsules for the four different preparation conditions (Figure 3d). The four release profiles show a similar trend that can be visually separated into three regions. Based on the observation during the release tests, we separate each release curve into three regimes: early diffusion (*R*1), bursting (*R*2), and postrelease (*R*3) regimes. In the early diffusion (*R*1) regime, the nanoemulsion droplets in the capsules (diameter ≈ 58 nm) slowly diffuse out through the shell yielding nanodroplets in the release medium (diameter ≈ 58 nm) detected by DLS. The droplet diffusion through the alginate shell can be explained by the swelling of the alginate hydrogels in a large volume of calcium‐free release medium, which expands the mesh size.^[^ ^46^ ^]^ During the *R*1 regime, the calcium alginate gel softens and weakens because the calcium ions are gradually displaced by sodium ions.^[^ ^47^ ^]^ When the alginate gel is weak enough, a crack forms on the shell and the nanoemulsion starts releasing. The onset of a bursting event leads to a transition of the release profile from a flat line into the region of a shaper slope, which indicates the time point at which the release process enters the bursting (*R*2) regime. In this regime, capsules randomly burst with the nanoemulsion cargo then quickly released into solution. For a single bursting event for a capsule, the nanoemulsion cargo can be released in a few minutes, followed by the remaining capsule dissolving completely in another few minutes. After all the bursting events are finished, the release process enters the postrelease (*R*3) regime. In the last regime, the absorbance signal reaches a saturated value indicating complete release of the cargo. The proposed release mechanism is also supported by the optical images of the capsules taken at different time points during a release process (Figure 3e–h). From Figure 3e,f, the shell thickness remains similar while the shell contrast decreases as the ion displacement proceeds for a sufficient amount of time. This indicates that the hydrogel degradation process is uniform across the shell and can be characterized as bulk erosion.^[^ ^48^ ^]^ For the bursting capsule, a crack forms on the shell, and the osmotic pressure between the two sides of the capsule drives the nanoemulsion to release out (Figure 3g). Because of the large viscosity difference, the nanoemulsion is released as a viscous jet and then gradually diffuses away. The empty capsule after the release can exist for a few minutes before complete degradation (Figure 3h).

### 1.4 Quantitative Analyses on the Release Profiles and Stability Tests

**Figure 4:**
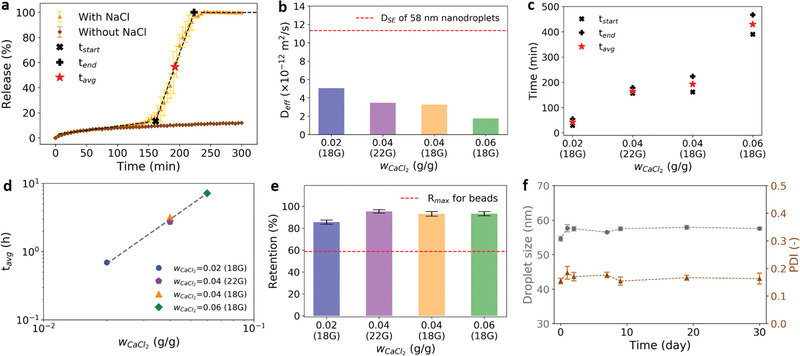
Quantitative analysis of release profiles and nanoemulsion stability tests. a) Comparison of release profiles of capsules ($w_{\mathrm{CaC}l_{2}}$= 0.04 and 18 gauge dispensing tip) at 37 °C in a saline solution (with 0.9 wt% NaCl) with that in deionized water (without NaCl). Three different regimes of the release profile in the NaCl bath are regressed with linear lines to determine the start (*t*_start_), average (*t*_avg_), and end (*t*_end_) time points in the bursting regime (*R*2). b) Effective diffusivities (*D*_eff_) of the nanoemulsion droplets (and micelles) through the alginate shell in regime *R*1 for different preparation conditions. The red dotted line is the diffusivity of free nanodroplets (diameter = 58 nm) in 37 °C water determined from the Stokes–Einstein equation. c) Burst time of capsules for different preparation conditions. d) Correlation of average burst time with $w_{\mathrm{CaC}l_{2}}$. e) Retention of the nanoemulsion suspension in capsules for different preparation conditions. The red dotted line is the highest retention rate achieved in nanoemulsion‐loaded alginate beads prepared from the same nanoemulsion. f) Nanoemulsion stability inside capsules sampled at various times over a month.

The early diffusion (*R*1) regime involves both the diffusion and shell degradation. To deconvolute these two effects, a control experiment with no shell degradation is done in a 37 °C deionized water bath (sodium‐free environment). The release profile for the control experiment (brown curve in **Figure** **4**a) closely follows the orange (saline bath) curve in the early diffusion (*R*1) regime, which indicates that the ion displacement process is slow and only begins to have a minor effect on the release in the late *R*1 regime. To quantify the *R*1 regime, the effective diffusivity (*D*_eff_) of the dispersed phase in the alginate shell for each condition is estimated (Figure 4b, complete results in Figure S7a–d in the Supporting Information). For typical hydrogels, the *D*_eff_ is inversely correlated with the crosslinking density.^[^ ^49^ ^]^ The *D*_eff_ in Figure 4b is observed to decrease with increasing $w_{\mathrm{CaC}l_{2}}$, which can be explained by a more abundant calcium source leading to a higher crosslinking density. To quantitatively identify the bursting (*R*2) regime for a dissolution profile, each regime of the profile is first fitted with a line. The two intersection points between the first two fitted lines (×) and the last two fitted lines (+) are defined as the start (*t*_start_) and end (*t*_end_) bursting time points. The time span between the *t*_start_ and *t*_end_ is characterized as the bursting regime in which bursting events can happen randomly in individual capsules to contribute to the increase in nanoemulsion released. The average bursting time (*t*_avg_) for the multiple bursting events is then determined by averaging *t*_start_ and *t*_end_ (Figure 4a; additional data in Figure S7e–h in the Supporting Information). The release behavior caused by bursting events can be fit by a cumulative distribution function (Figure S8, Supporting Information), revealing that the bursting events are statistically random and follow a normal distribution centered at *t*_avg_. For the same dispensing tip size, increasing the $w_{\mathrm{CaC}l_{2}}$ leads to a longer average bursting time and a larger standard deviation of the fitted distribution. The longer average bursting time is attributed to the combined effects of the higher crosslinking density and the thicker shell. Because both the crosslinking density and shell thickness increase with the increasing $w_{\mathrm{CaC}l_{2}}$, we assume a power‐law function of $w_{\mathrm{CaC}l_{2}}$ to empirically fit *t*_avg_. The power‐law fitting can approximately describe the trend between $w_{\mathrm{CaC}l_{2}}$ and *t*_avg_ with an exponent of 2.2 (Figure 4d), which can be used as the simple design rule for engineering the bursting time of the alginate capsules.

For comparison, we also prepared nanoemulsion‐loaded alginate beads by dripping alginate naonemulsions into a calcium bath (Figure S9a, Supporting Information). The alginate naonemulsion solutions containing alginate but lacking calcium ions were prepared with the same oil phase, surfactants, and weight fraction as the calcium nanoemulsions. The alginate nanoemulsions with different alginate concentrations show droplet sizes between 50 and 55 nm (Figure S9b, Supporting Information), which are very similar to those of the calcium nanoemulsions and allow us to compare the two carrier systems with parity. One of the main differences between the two systems is the retention rates of the naonemulsion suspension in the formed particles. The capsule gelation bath remains optically clear throughout the gelation process, while in contrast a severe leakage is observed in the bead gelation bath with the transparent bath turning bluish. Because the disparity between the droplet size and alginate mesh size is within an order of magnitude, excessive oil droplets and surfactants are likely to hinder the alginate crosslinking. The severe leakage of the nanoemulsion from the alginate beads is reflected by a low retention rate, which is about 58.8% for the 4% w/v alginate beads and even lower for lower alginate concentrations (Figure S10c, Supporting Information). In contrast, the alginate capsules display retention rates higher than 85% for all the conditions (Figure 4e). For the $w_{\mathrm{CaC}l_{2}}$ higher than 0.04, the retention rates are all above 93%, indicating the effective encapsulation of the nanoemulsion. Detailed calculations for the retention rates of the two carrier systems are tabulated in Tables S2 and S3 (Supporting Information). Figure 4f shows the droplet size of the nanoemulsion in capsules over time. The droplet size remains stably around 58 nm over a month with the PDI value lying between 0.1 and 0.2. Because of the uniform distribution and small size, effects of Ostwald ripening, flocculation, and coalescence are minimal.

### 1.5 Versatility and Extended Applications for Nanoemulsion‐Loaded Capsules

**Figure 5:**
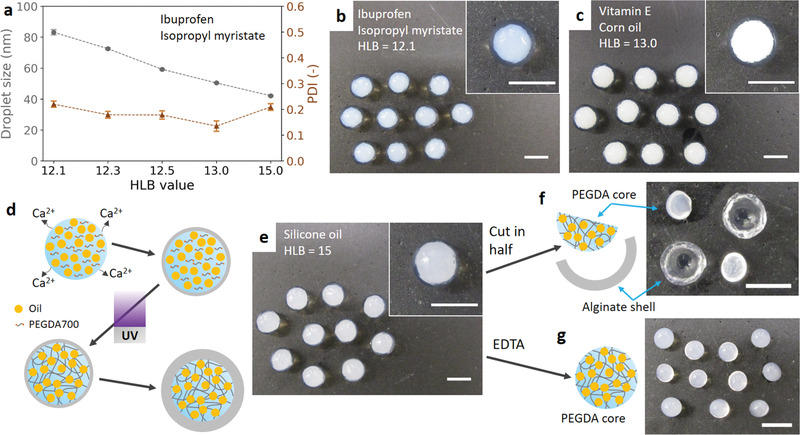
Versatility of the approach to prepare capsules with different nanoemulsion sizes, various active chemicals, and chemically crosslinked cores. a) Relationship between nanoemulsion droplet size and HLB value. The dispersed phase is ibuprofen‐loaded isopropyl myristate. b) Optical image of nanoemulsion‐loaded capsules prepared from the calcium nanoemulsion with the HLB value of 12.1 and the dispersed phase of ibuprofen‐loaded isopropyl myristate (droplet size = 83.15 ± 1.70 nm). c) Optical image of nanoemulsion‐loaded capsules prepared from the calcium nanoemulsion with the HLB value of 13 and the dispersed phase of vitamin E‐loaded corn oil (droplet size = 135.04 ± 1.57 nm). d) Schematic diagram of the preparation of core–shell hydrogels with a nanoemulsion‐laden, chemically crosslinked PEGDA core. e) Optical image of the core–shell hydrogels with a nanoemulsion‐laden solid core. The nanoemulsion has an HLB value of 15 and the dispersed phase of silicone oil (droplet size = 41.60 ± 0.73 nm). f) Optical image of core–shell hydrogels after being cut in half and physically extracting the core. g) Optical image of PEGDA hydrogel cores collected by dissolving alginate shells in an EDTA solution. All capsules in this figure are prepared with $w_{\mathrm{CaC}l_{2}}$= 0.04 and an 18 gauge dispensing tip. All scale bars are 5 mm.

To enhance the versatility of the capsule systems, different droplet sizes are engineered by varying the ratio of the two surfactants (**Figure** **5**a). Decreasing emulsion droplet size has been reported to increase the intestinal absorption of active ingredients.^[^ ^50^ ^]^ As the surfactant mixture becomes more hydrophilic (increased HLB value), the droplet size decreases. The calcium nanoemulsion with a droplet size of 83 nm is chosen as an example to be encapsulated in alginate capsules for demonstration (Figure 5b). Because of the larger droplet size, the nanoemulsion liquid core has a bluish‐white opacity. In addition to the ibuprofen‐loaded isopropyl myristate, the encapsulation technique can also be applied to other combinations of active ingredients and oils. Figure 5c shows the encapsulation of a nanoemulsion prepared from a vitamin E‐loaded corn oil. The nanoemulsion has a droplet size of 135 nm, thereby leading to an opaque white appearance of the nanoemulsion liquid core due to light scattering. To further extend the applications, core–shell hydrogel particles with nanoemulsion‐laden chemically crosslinked hydrogel cores are developed (Figure 5d). A UV‐crosslinkable nanoemulsion is prepared using a 25 wt% PEGDA700 aqueous solution and a silicone oil. After the addition of CaCl_2_, the nanoemulsion shows a dual gelation capability for both the alginate and UV light. The alginate thin shell is instantaneously formed when the nanoemulsion is dripped into an alginate bath. The bath is then exposed to a UV light to fully crosslink the nanoemulsion liquid cores into the hydrogels with silicone oil droplets locally locked and embedded in the inner hydrogel matrices. The alginate shell continues growing in the bath until the calcium ions are depleted. The as‐prepared core–shell hydrogels are uniform in size (Figure 5e), and the UV‐crosslinked inner core is further examined by cutting the hydrogel in half (Figure 5f) and by dissolving the alginate shells in an ethylenediaminetetraacetic acid (EDTA) solution (Figure 5g). The inner core is solid and can be easily detached from the alginate shell, indicating that the UV‐crosslinking is effective and the two hydrogel networks are not interpenetrating. Moreover, the shell thickness (0.745 ± 0.051 mm) is almost identical to the canonical condition using the same concentration of CaCl_2_ ($w_{\mathrm{CaC}l_{2}}$= 0.04), demonstrating that the shell thickness is not affected by the type of nanoemulsion. In prior studies, introducing additional hydrogel shells around UV‐crosslinked hydrogels generally requires multiple steps: first preparing the core particles and encapsulating them in another polymeric shell.^[^ ^51^ ^]^ The core and shell hydrogels have to be formed separately because the two crosslinkable precursors are miscible. In contrast, our double gelation technique enables the formation of additional alginate shells around UV‐crosslinked hydrogels in a single batch. The PEGDA700 molecules are crosslinked shortly after the nanoemulsion is dripped into the bath, minimizing the diffusion of PEGDA700 molecules into the alginate bath and creating a well‐defined boundary between the core and shell regions (Figure 5f). The introduction of additional hydrogel shells around the nanoemulsion‐laden hydrogels can find use in various applications, including controlled lipid digestion^[^ ^25^, ^26^ ^]^ and tunable delayed release for confined crystallization.^[^ ^3^, ^28^, ^29^, ^30^ ^]^

## 2 Conclusions

We have developed versatile and functional capsule systems for controlled delivery of nanoemulsions that contain lipophilic active ingredients. For the formation of spherical capsules, we leverage the intrinsically high viscosity of the nanoemulsions with only a minor modulation of the viscosity of the continuous aqueous phase through added sucrose. The large viscosity prevents the dripped droplets from deformation due to the drag force exerted by the surrounding alginate solution. The capsule shell thickness and inner core size are easily controlled by varying the calcium concentration and the dispensing tip size, respectively. To investigate the controlled release of the nanoemulsions from the capsules, we developed a facile method to quantify the release of the nanoemulsions through direct UV–vis measurements in the release vessel. The release profiles of the capsules for different preparation conditions follow a similar trend, and three different release regimes are defined. The nanodroplets first diffuse out slowly through the alginate shell, followed by rapid release due to capsule bursting in a saline solution. The release regimes were quantified to determine the effective nanoemulsion diffusivity through the shell and the bursting time for the capsules. A simple power‐law equation was developed as a design rule to correlate the bursting time and the calcium concentration. The results show that the bursting time of the capsules can be easily engineered by varying the calcium concentration in the nanoemulsion suspension. The capsules and their contents were very stable, with nanoemulsion droplet size remaining nearly constant over a month of storage. To demonstrate the generality of the approach, nanoemulsion‐loaded capsule systems were fabricated with other combinations of active ingredients and oils. In addition, UV‐crosslinkable precursors were added into the nanoemulsion solution for the preparation of core–shell hydrogel particles with the oil nanodroplets embedded in the inner chemically crosslinked hydrogel cores. Overall, the versatile capsule systems show great promise for designing carriers with controlled release of functional nanoemulsions, which can find use in a wide range of applications in the food, pharmaceutical, and cosmetic industries.

## 3 Experimental Section

##### Materials

Sodium alginate (≈39% in guluronic acid blocks, *M*_w_ ≈ 100 kDa), Span 80 (sorbitan monooleate), Tween 80 (polysorbate), isopropyl myristate (≥98%), calcium chloride (CaCl_2_), sodium chloride (NaCl), Nile red, vitamin E (*α*‐tocopherol, ≥ 99.5%), corn oil, poly(ethylene glycol) diacrylate (PEGDA, *M*_n_ = 700 g mol^−1^), silicone oil, and photoinitiator 2‐hydroxy‐2‐methyl‐1‐phenyl‐propan‐1‐one (Darocur 1173) were purchased from Sigma–Aldrich. Ibuprofen (99%, ACROS Organics) was purchased from Fisher Scientific to be used as an API. Sucrose (ultrapure) was purchased from VWR Life Science.

##### Nanoemulsion Synthesis

Nanoemulsions were prepared using a low‐energy method (phase inversion approach) at ambient temperature. In this study, two nanoemulsion systems (calcium nanoemulsions and alginate nanoemulsions) were developed for the preparation of alginate capsules and alginate beads, respectively. The two systems were composed of the same oil phase and surfactant. The oil (dispersed) phase was isopropyl myristate loaded with ibuprofen (100 mg mL^−1^), and the surfactant was a mixture of Tween 80 and Span 80 with an HLB value of 13. The HLB value for a mixture of Tween 80 and Span 80 was calculated by HLB = 4.3*x*_w_ + 15(1 − *x*_w_), where *x_w_* is the weight fraction of Span 80 in the mixture. Span 80 and Tween 80 had HLB values of 4.3 and 15, respectively, which represent oleophilic and hydrophilic surfactants, respectively. To engineer the nanoemulsion droplet sizes, calcium nanoemulsions were prepared with surfactant mixtures of different HLB values. For the preparation of calcium nanoemulsions, a 3 g sucrose aqueous solution (25 wt%) was slowly dripped into a mixture of 1 g API‐loaded oil and 1 g surfactant at a magnetic stirring rate of 1000 rpm. After stirred for another 10 min at the same speed, the nanoemulsion was added to CaCl_2_ (0.1/0.2/0.3 g). The resulting calcium concentrations ($w_{\mathrm{CaC}l_{2}}$) were 0.02, 0.04, and 0.06 g g^−1^ nanoemulsion, respectively. For the alginate nanoemulsion, a 3 g alginate aqueous solution (1/2/4% w/v) was slowly dripped into a mixture of 1 g API‐loaded oil and 1 g surfactant at a magnetic stirring rate of 1000 rpm. After another 10 min of stirring at the same speed, the alginate nanoemulsion was obtained.

##### Dynamic Light Scattering

The droplet size and PDI of a nanoemulsion were measured by DLS (Brookhaven NanoBrook 90Plus PALS) operated at a fixed scattering angle of 90° and a temperature of 25 °C. The sample was prepared by diluting 5 µL of the nanoemulsion solution with 3 mL deionized water in a cuvette. The dilution was performed to eliminate multiple scattering effects and ensure a consistent baseline. For each sample, five sets of 1 min measurements were done to determine the droplet size distribution.

##### Alginate Capsules for Nanoemulsion Encapsulation

The inverse gelation technique was used to prepare alginate capsules.^[^ ^33^ ^]^ The gelation bath was a 200 mL 1% w/v CaCl_2_ solution with 0.1% w/v Tween 80 added to lower the surface tension. For nanoemulsion encapsulation, 3 mL of a calcium nanoemulsion was loaded into a syringe and dripped into the bath at a dripping height of 10 cm. The stirring rate was maintained at 350 rpm to enhance the mass transfer of calcium ions and prevent the capsules in close contact from sticking together due to the fast gelation process. An 18G (Nordson EFD Optimum, inner diameter = 0.84 mm, outer diameter = 1.27 mm, length = 12.7 mm) stainless steel dispensing tip was used to drip the calcium nanoemulsions with three different calcium concentrations. For the calcium concentration ($w_{\mathrm{CaC}l_{2}}$) of 0.04 g g^−1^ nanoemulsion, 22 gauge (22G, Nordson EFD Optimum, inner diameter = 0.41 mm, diameter = 0.72 mm, and length = 12.7 mm) stainless steel dispensing tip was also used to prepare smaller capsules for comparison. After all the nanoemulsion in the syringe was dripped into the bath, the bath was stirred for another 1.5 h. Before the capsules were collected, the alginate bath was diluted fourfold by adding 600 mL deionized water to quench the gelation process. The collected capsules were then washed by deionized water briefly and incubated in a 2% w/v CaCl_2_ solution at a stirring rate of 350 rpm for 15 min. Finally, the alginate capsules were rinsed with deionized water again to remove excess CaCl_2_ and stored in the refrigerator overnight before release tests.

##### Alginate Beads for Nanoemulsion Encapsulation

The external gelation technique was used to prepare alginate beads.^[^ ^33^ ^]^ A 3 mL alginate nanoemulsion was loaded into a syringe and dripped into a 200 mL 2% w/v CaCl_2_ solution (containing 0.1% w/v Tween 80) at a dripping height of 10 cm. The stirring rate was maintained at 100 rpm to enhance the mass transfer of the calcium ions. An 18G stainless steel dispensing tip was used for dripping the alginate nanoemulsion with three different alginate concentrations. After all the nanoemulsion in the syringe was dripped into the bath, the CaCl_2_ concentration of the bath was increased to 4% w/v by adding more CaCl_2_ into the bath, and the beads were stirred for another 2 h to ensure complete crosslinking. Finally, the alginate beads were rinsed with deionized water to remove excess CaCl_2_ and stored in the refrigerator overnight before the release test.

##### Particle Size and Shell Thickness Analyses

The images of the as‐prepared nanoemulsion carriers (capsules and beads) were captured from the top view using a digital camera (Canon PowerShot ELPH 190 IS). ImageJ was used as an image processing tool to characterize the area (*A*) as well as the maximum and minimum Feret diameters (*d*_max_ and *d*_min_). With these parameters, the carrier outer radius (*r*_carrier_) and sphericity factor (SF) can be calculated as follows

(1) $$\begin{array}{c} r_{\mathrm{carrier}}=\sqrt{A/\pi } \end{array}$$

(2) $$\begin{array}{c} \mathrm{SF}\;=\;\frac{d_{\max}\;-\;d_{\min}}{d_{\max}\;+\;d_{\min}} \end{array}$$

To further determine the inner core radius and thickness of the alginate capsules, the capsules were cut in half, and their images were analyzed by ImageJ to determine the shell thickness (*h*_shell_). Finally, the core radius (*r*_core_) was obtained by subtracting the *h*_shell_ from the *r*_carrier_. For each condition, ten carriers were used for analyses.

##### Viscosity Measurement

TA Instruments DHR‐3 stress‐controlled rotational rheometer was used to measure the viscosity of the API‐loaded nanoemulsion and its continuous phase combinations. The rheometer was equipped with an upper‐cone geometry (diameter = 60 mm, cone angle = 1.004°, and truncated gap = 29 µm). Viscosities were measured by carrying out shear rate sweeps from 0.1 to 1000 s^−1^ at 20 °C. The equilibration time and averaging time were set to be 5 and 30 s.

##### UV–Vis Spectroscopy

A UV–vis spectrophotometer (Thermo Scientific NanoDrop One) was used to measure the absorbance spectra of two samples: bulk oil phase (100 mg ibuprofen mL^−1^ isopropyl myristate) and O/W nanoemulsion prepared from the oil phase. The light wavelength was swept from 150 to 850 nm. Pure isopropyl myristate and deionized water was used to determine the baseline for the bulk oil phase and the nanoemulsion, respectively. The characteristic absorbance peaks of the two samples were both at 230 nm.

##### Release Experiment

The in vitro release of API‐loaded nanoemulsions from the alginate capsules and beads was measured using a USP Dissolution Apparatus II (Agilent Technologies Varian VK 7025). A Cary 50 UV–vis spectrometer and an in situ probe set, which were integrated in the dissolution apparatus, automatically recorded the absorbance at a wavelength of 230 nm every minute. The release medium of 900 mL 0.9% w/v (0.154 m) saline was used to simulate physiological conditions because of the similar osmolarity to human body fluids. The operating temperature and paddle rotational speed were set at 37 °C and 75 rpm, respectively. Before release experiments, the UV–vis spectrometer was first calibrated with an API‐loaded nanoemulsion. The nanoemulsion was then added sequentially into the vessel with the addition mass and the corresponding absorbance recorded. For each release experiment, the number of nanoemulsion carriers (capsules or beads) was controlled so that about 500 mg optimal API‐loaded nanoemulsion was released from the carriers. The required numbers of carriers prepared from 18 and 22G needles were 40 and 70, respectively. All reported measurements were repeated three times under identical conditions and averaged values were reported.

##### Determination of Effective Diffusivity for the Early Diffusion (*R*1) Regime

Assuming a steady‐state diffusion in the radial direction through a spherical shell with inner (*r*_i_) and outer (*r*_o_) radii, the following mass transfer per time *n_A_* (mol s^−1^) was obtained

(3) $$\begin{array}{c} n_{A}=\frac{4\pi r_{i}r_{o}D_{\mathrm{eff}}\left(C_{A,\mathrm{in}}-\;C_{A,\mathrm{out}}\right)}{r_{o\;}-\;r_{i}}\; \end{array}$$

where *C*_A,in_ and *C*_A,out_ are the species concentrations of the capsule core and bath, and *D*_eff_ is the effective diffusivity of the species in the shell. Because the *D*_eff_ was determined for the early diffusion regime where most of the nanoemulsion still remains in the capsules, the equation was further approximated by setting *C*_A,in_ and *C*_A,out_ to be *C*_A,0_ (initial species concentration of the capsule core) and 0, respectively. Based on the fact that the cumulative release *R* reaches 100% for the complete release, the *C*_A,0_ can be further represented in the unit of *R* (%)

(4) $$\begin{array}{c} C_{A,0}=\frac{100\%\left(V_{b}+n_{c}V_{c}\right)}{n_{c}V_{c}}\; \end{array}$$

where *V*_b_ is the bath volume, *n*_c_ is the number of carriers (capsules) added into the release vessel, and *V*_c_ is the core volume for each capsule. In the early diffusion regime, the *R* increases linearly with time. Therefore, the *n*_A_ can be approximated as

(5) $$\begin{array}{c} n_{A}=\frac{\frac{\Delta R}{\Delta t}V_{b}}{n_{c}}\; \end{array}$$

where Δ*R*/Δ*t* is the slope fitted from the *R* (%) in this regime. Combining the above three equations, an expression was obtained for the *D*_eff_

(6) $$\begin{array}{c} D_{\mathrm{eff}}=\frac{\frac{\Delta R}{\Delta t}V_{b}\left(r_{o}-r_{i}\right)}{4\pi r_{i}r_{o}n_{c}C_{A,0}}\; \end{array}$$

The diffusivity of the free nanodroplets in the release medium (37 °C 0.9% w/v saline solution) can be estimated by the Stokes–Einstein equation (Equation (7)) as an upper bound of the *D*_eff_

(7) $$\begin{array}{c} D_{\mathrm{SE}}=\frac{k_{B}T}{6\pi \eta_{w}r_{\mathrm{NE}}}\; \end{array}$$

where *k*_B_ is the Boltzmann constant (1.38 × 10^−23^ J K^−1^), *T* is the bath temperature (310.15 K), *η*_w_ is the water viscosity at 37 °C (0.69 mPa s), and *r*_NE_ is the nanoemulsion droplet radius (≈29 nm). The corresponding *D*_SE_ is 1.13 × 10^–11^ m^2^ s^−1^.

##### Retention Rate Estimation

The retention rate of nanoemulsion carriers was estimated from the result of the release test. Theoretically, the optimal nanoemulsion mass *m*_opt _ (for 100% retention) added in the vessel was estimated by

(8) $$\begin{array}{c} m_{\mathrm{opt}\;}=n_{c}m_{d} \end{array}$$

where *n*_c_ is the number of carriers (capsules or beads) added into the release vessel, *m*_d_ is the mass of each nanoemulsion droplet dripped into the gelation bath through a syringe for encapsulation. After the release experiment, the actual nanoemulsion mass in the release vessel was determined as

(9) $$\begin{array}{c} m_{\mathrm{act}}=I_{\mathrm{sat}}/s_{\mathrm{cal}} \end{array}$$

where *I*_sat_ is the absorbance of saturated bath when all the nanoemulsion was released from the carriers and *s*_cal_ is the slope of the calibration curve (Figure 3c) for the mass and absorbance. The retention (*R*_t_) is expressed as *R*_t_ = *m*_act_ /*m*_opt_.

##### Vitamin E‐Loaded Nanoemulsion‐Encapsulated Capsule

A 3 g of sucrose aqueous solution (25 wt%) was slowly dripped into a mixture of 1 g of vitamin E‐loaded corn oil (20 wt% vitamin E) and 1 g of surfactants (HLB = 13) at a magnetic stirring rate of 1000 rpm. After another 5 min of stirring, the resulting pre‐emulsion was ultrasonicated at 30% amplitude in an ultrasonicator with a 24 mm diameter horn (from Cole Parmer) at a frequency of 20 kHz for 5 min. Finally, 0.2 g of CaCl_2_ was added into the nanoemulsion. The naonemulsion was encapsulated in alginate capsules following the same inverse gelation procedure as described above.

##### Core–Shell Hydrogel with a Nanoemulsion‐Laden Solid Core

A 3 g PEGDA700 aqueous solution (25 wt%) was slowly dripped into a mixture of 1 g silicone oil and 1 g surfactant (HLB = 15, pure Tween 80) at a magnetic stirring rate of 1000 rpm. After another 5 min stirring, the resulting pre‐emulsion was ultrasonicated at 20% amplitude in an ultrasonicator with a 24 mm diameter horn (from Cole Parmer) at a frequency of 20 kHz for 15 min. Finally, 0.2 g of CaCl_2_ and 50 µL of photoinitiator (Darocur 1173) were added into the nanoemulsion. To encapsulate the UV‐crosslinkable calcium nanoemulsion, the same inverse gelation technique was applied. The only difference was that the alginate bath was exposed to a UV lamp (365 nm, 1.3 W) for the first 30 min of the gelation period.

## Conflict of Interest

The authors declare no conflict of interest.

## Supporting information

Supporting Information

## References

[1] C. Lovelyn , A. A. Attama , J. Biomater. Nanobiotechnol. 2011, 02, 626.

[2] H. Chen , C. Khemtong , X. Yang , X. Chang , J. Gao , Drug Discovery Today 2011, 16, 354. 20206289 10.1016/j.drudis.2010.02.009

[3] A. Z. M. Badruddoza , A. Gupta , A. S. Myerson , B. L. Trout , P. S. Doyle , Adv. Ther. 2018, 1, 1700020.

[4] S. M. Hashemnejad , A. Z. M. Badruddoza , B. Zarket , C. Ricardo Castaneda , P. S. Doyle , Nat. Commun. 2019, 10, 2749. 31227703 10.1038/s41467-019-10749-1 PMC6588569

[5] D. J. McClements , Soft Matter 2011, 7, 2297.

[6] D. J. McClements , J. Rao , Crit. Rev. Food Sci. Nutr. 2011, 51, 285. 21432697 10.1080/10408398.2011.559558

[7] T. Loftsson , M. E. Brewster , J. Pharm. Pharmacol. 2010, 62, 1607. 21039545 10.1111/j.2042-7158.2010.01030.x

[8] A. Gupta , H. B. Eral , T. A. Hatton , P. S. Doyle , Soft Matter 2016, 12, 2826. 26924445 10.1039/c5sm02958a

[9] Y. Singh , J. G. Meher , K. Raval , F. A. Khan , M. Chaurasia , N. K. Jain , M. K. Chourasia , J. Controlled Release 2017, 252, 28. 10.1016/j.jconrel.2017.03.008 28279798

[10] H. Yu , Q. Huang , J. Agric. Food Chem. 2012, 60, 5373. 22506728 10.1021/jf300609p

[11] L. Salvia‐Trujillo , R. Soliva‐Fortuny , M. A. Rojas‐Graü , D. J. McClements , O. Martín‐Belloso , Annu. Rev. Food Sci. Technol. 2017, 8, 439. 28125342 10.1146/annurev-food-030216-025908

[12] S. M. Jafari , D. J. McClements , Nanoemulsions: Formulation, Applications, and Characterization, Elsevier Inc., London 2018, p. 435.

[13] L. L. Augsburger , S. W. Hoag , Pharmaceutical Dosage Forms: Capsules, CRC Press, Boca Raton, FL 2017.

[14] E. Amstad , ACS Macro Lett. 2017, 6, 841.

[15] S. S. Datta , A. Abbaspourrad , E. Amstad , J. Fan , S. H. Kim , M. Romanowsky , H. C. Shum , B. Sun , A. S. Utada , M. Windbergs , S. Zhou , D. A. Weitz , Adv. Mater. 2014, 26, 2205. 24615984 10.1002/adma.201305119

[16] Q. Ma , Y. Song , J. W. Kim , H. S. Choi , H. C. Shum , ACS Macro Lett. 2016, 5, 666. 10.1021/acsmacrolett.6b00228 35614670

[17] K. J. Tangso , H. Patel , S. Lindberg , P. G. Hartley , R. Knott , P. T. Spicer , B. J. Boyd , ACS Appl. Mater. Interfaces 2015, 7, 24501. 26457761 10.1021/acsami.5b05821

[18] G. Etienne , I. L. H. Ong , E. Amstad , Adv. Mater. 2019, 31, 1808233. 10.1002/adma.201808233 31081156

[19] J. Li , D. J. Mooney , Nat. Rev. Mater. 2016, 1, 16071. 29657852 10.1038/natrevmats.2016.71 PMC5898614

[20] C. C. Lin , A. T. Metters , Adv. Drug Delivery Rev. 2006, 58, 1379. 10.1016/j.addr.2006.09.004 17081649

[21] K. Y. Lee , D. J. Mooney , Prog. Polym. Sci. 2012, 37, 106. 22125349 10.1016/j.progpolymsci.2011.06.003 PMC3223967

[22] A. D. Augst , H. J. Kong , D. J. Mooney , Macromol. Biosci. 2006, 6, 623. 16881042 10.1002/mabi.200600069

[23] F. Zhao , D. Yao , R. Guo , L. Deng , A. Dong , J. Zhang , Nanomaterials 2015, 5, 2054. 28347111 10.3390/nano5042054 PMC5304774

[24] D. Jagadeesan , I. Nasimova , I. Gourevich , S. Starodubtsev , E. Kumacheva , Macromol. Biosci. 2011, 11, 889. 21504069 10.1002/mabi.201100045

[25] Y. Li , M. Hu , Y. Du , H. Xiao , D. J. McClements , Food Hydrocolloids 2011, 25, 122. 10.1016/j.foodhyd.2011.08.017 PMC3367464 22685367

[26] M. N. Corstens , C. C. Berton‐Carabin , P. T. Elichiry‐Ortiz , K. Hol , F. J. Troost , A. A. M. Masclee , K. Schroën , J. Funct. Foods 2017, 34, 319.

[27] E. S. Chan , Carbohydr. Polym. 2011, 84, 1267.

[28] A. Z. M. Badruddoza , P. D. Godfrin , A. S. Myerson , B. L. Trout , P. S. Doyle , Adv. Healthcare Mater. 2016, 5, 1960. 10.1002/adhm.201600266 27249402

[29] T. Domenech , P. S. Doyle , Chem. Mater. 2020, 32, 498.

[30] H. B. Eral , M. O'Mahony , R. Shaw , B. L. Trout , A. S. Myerson , P. S. Doyle , Chem. Mater. 2014, 26, 6213.

[31] B. Zeeb , A. H. Saberi , J. Weiss , D. J. McClements , Food Hydrocolloids 2015, 50, 27.

[32] H. Z. An , M. E. Helgeson , P. S. Doyle , Adv. Mater. 2012, 24, 3838. 22451097 10.1002/adma.201200214

[33] J. Y. Leong , W. H. Lam , K. W. Ho , W. P. Voo , M. F. X. Lee , H. P. Lim , S. L. Lim , B. T. Tey , D. Poncelet , E. S. Chan , Particuology 2016, 24, 44.

[34] A. Gupta , A. Z. M. Badruddoza , P. S. Doyle , Langmuir 2017, 33, 7118. 28654749 10.1021/acs.langmuir.7b01104

[35] E. Csányi , B. Sütő , S. Berkó , G. Kozma , Á. Kukovecz , M. Budai‐Szűcs , G. Erős , L. Kemény , A. Sztojkov‐Ivanov , R. Gaspar , Int. J. Nanomedicine 2016, 1201 10.2147/ijn.s99198. 27099487 PMC4821397

[36] D. J. McClements , Soft Matter 2012, 8, 1719.

[37] A. Blandino , M. Macías , D. Cantero , Process Biochem. 2001, 36, 601.

[38] A. Blandino , M. Macías , D. Cantero , J. Biosci. Bioeng. 1999, 88, 686. 16232687 10.1016/s1389-1723(00)87103-0

[39] J. F. Brady , J. Chem. Phys. 1993, 99, 567.

[40] D. Quemada , C. Berli , Adv. Colloid Interface Sci. 2002, 98, 51. 12061712 10.1016/s0001-8686(01)00093-8

[41] T. F. Tadros , Adv. Colloid Interface Sci. 1996, 68, 97.

[42] A. Amani , P. York , H. De Waard , J. Anwar , Soft Matter 2011, 7, 2900.

[43] K. Haldar , S. Chakraborty , J. Colloid Interface Sci. 2018, 528, 156. 29852345 10.1016/j.jcis.2018.05.078

[44] T. G. Mason , J. N. Wilking , K. Meleson , C. B. Chang , S. M. Graves , J. Phys.: Condens. Matter 2006, 18, R635.

[45] E. S. Chan , B. B. Lee , P. Ravindra , D. Poncelet , J. Colloid Interface Sci. 2009, 338, 63. 19604515 10.1016/j.jcis.2009.05.027

[46] B. H. Lee , B. Li , S. A. Guelcher , Acta Biomater. 2012, 8, 1693. 22306825 10.1016/j.actbio.2012.01.012 PMC3314144

[47] L. Rolland , E. Santanach‐Carreras , T. Delmas , J. Bibette , N. Bremond , Soft Matter 2014, 10, 9668. 25363731 10.1039/c4sm02012j

[48] F. Von Burkersroda , L. Schedl , A. Göpferich , Biomaterials 2002, 23, 4221. 12194525 10.1016/s0142-9612(02)00170-9

[49] C. C. Lin , K. S. Anseth , Pharm. Res. 2009, 26, 631. 19089601 10.1007/s11095-008-9801-2 PMC5892412

[50] B. D. Tarr , S. H. Yalkowsky , Pharm. Res. 1989, 6, 40. 2717516 10.1023/a:1015843517762

[51] S. Seiffert , J. Thiele , A. R. Abate , D. A. Weitz , J. Am. Chem. Soc. 2010, 132, 6606. 20397695 10.1021/ja102156h

