# Supplementary material for: Nanoemulsion‐Loaded Capsules for Controlled Delivery of Lipophilic Active Ingredients
Source: Adv Sci (Weinh). 2020 Aug 28;7(20):2001677. doi: 10.1002/advs.202001677 (PMC7578884; doi:10.1002/advs.202001677)
Supplement: Supplementary file 1 — Supporting Information [file ADVS-7-2001677-s001.pdf]

© 2020 Wiley-VCH GmbH

ADVANCED SCIENCE
Open Access

Supporting Information

for *Adv. Sci.*, DOI: 10.1002/advs.202001677

Nanoemulsion-Loaded Capsules for Controlled Delivery of Lipophilic Active Ingredients

*Liang-Hsun Chen, Li-Chiun Cheng, and Patrick S. Doyle**

Supporting Information

Nanoemulsion-loaded capsules for controlled delivery of lipophilic active ingredients

*Liang-Hsun Chen, Li-Chiun Cheng, Patrick S. Doyle**

S.1. Conditions for the Formation of Spherical Capsules

When a liquid (calcium precursor) droplet penetrates into a gelation bath, the viscous and surface tension forces have to overcome the impact and drag forces to ensure the formation of spherical capsules. The Ohnesorge number (Oh) can be used to measure the relative importance of inertial, viscous, and surface tension stresses.[1]

$$Oh = \frac{\eta}{\sqrt{\rho d \gamma}} \tag{S1}$$

where η is the viscosity of the liquid, ρ is the density of the liquid, d is the diameter of the liquid droplet, and γ is the surface tension of the liquid.

For alginate bead formation (dripping an alginate solution into a $CaCl_2$ solution), prior work found that the Oh of alginate solution had to exceed 0.24 (a critical value) to overcome the impact and drag forces for forming spherical beads.[1] However, for alginate capsule formation (dripping a calcium precursor into an alginate bath), the critical value of Oh should be higher than 0.24 because of the large viscosity of the alginate bath. In this work, the calcium nanoemulsion has a high viscosity (301 mPa-s) and a low surface tension (35.7 mN/m). Both of these properties favor a large value of Oh, and the Oh for dripping the nanoemulsion with 18G and 22G dispensing tips are 0.79 and 0.89, respectively (**Table S1**).

Table S1. Determination of Oh numbers for dripping the nanoemulsion with 18G and 22G dispensing tips (d_{drop} is approximated by $2r_{core}$).

	η_{NE} (mPa-s)	ρ_{NE} (g/cm^3)	d_{drop} (mm)	γ_{NE} (mN/m)	Oh (-)
18G	301	1.09	3.75	35.7	0.79
22G	301	1.09	2.97	35.7	0.89

S.2. Viscosity-Shear Rate Flow Curves

Figure S1 shows the viscosity-shear rate flow curves for the different fluids measured in this study. The water, 25 wt.% sucrose solution, and 1% w/v alginate solution are Newtonian over the measured shear rate range. The sucrose-surfactants mixture is a complex fluid and displays minor shear thinning. The calcium nanoemulsion remains Newtonian up to a shear rate of 100 s^{-1} and transitions to a moderate shear thinning region at higher shear rates.

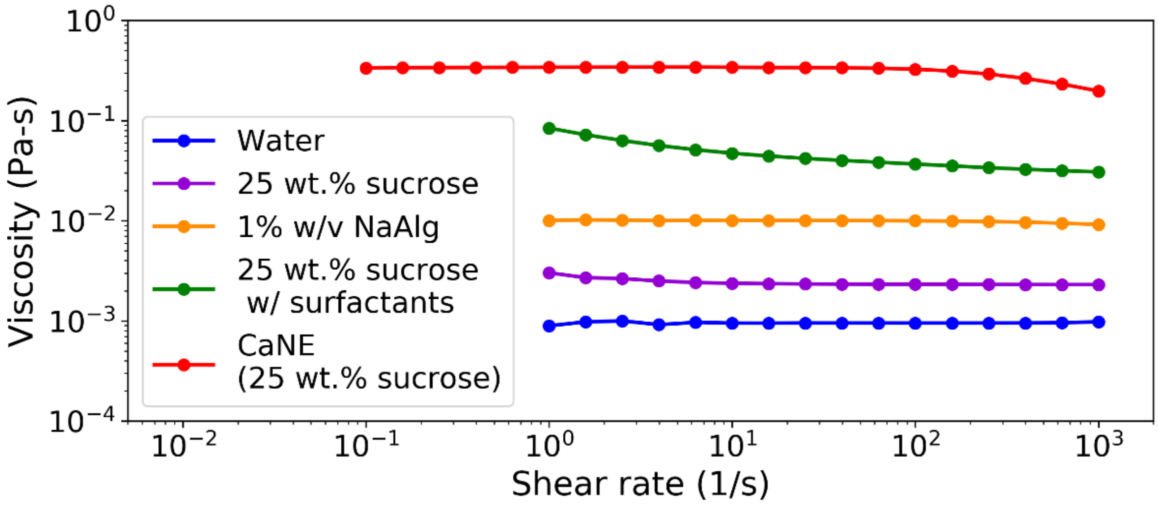

Figure S1. Viscosity-shear rate flow curves at 20°C for water, 25 wt.% sucrose and 1% w/v alginate solution, 25 wt.% sucrose with surfactants, and calcium nanoemulsion.

S.3. Optical Image of Nanoemulsion-Loaded Capsules

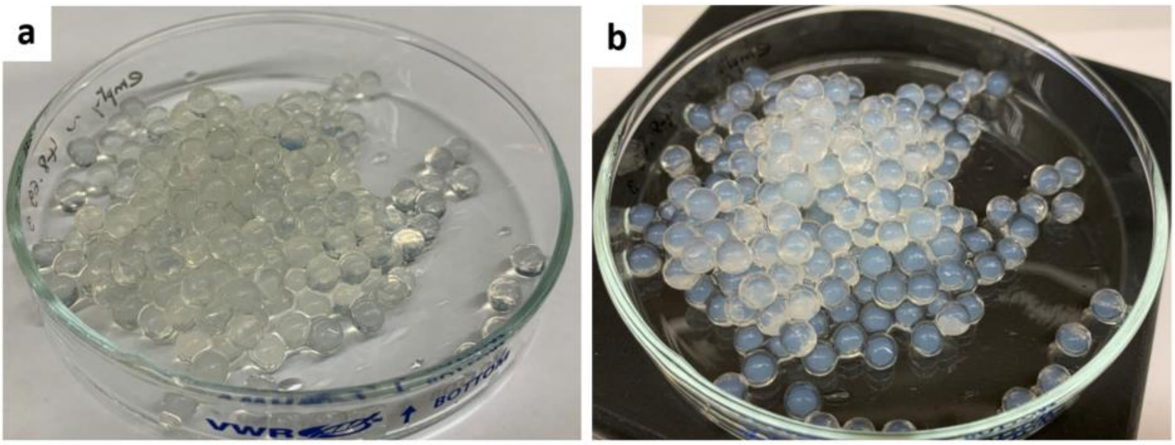

Figure S2. a-b) Optical images of capsules against a) white and b) black backgrounds.

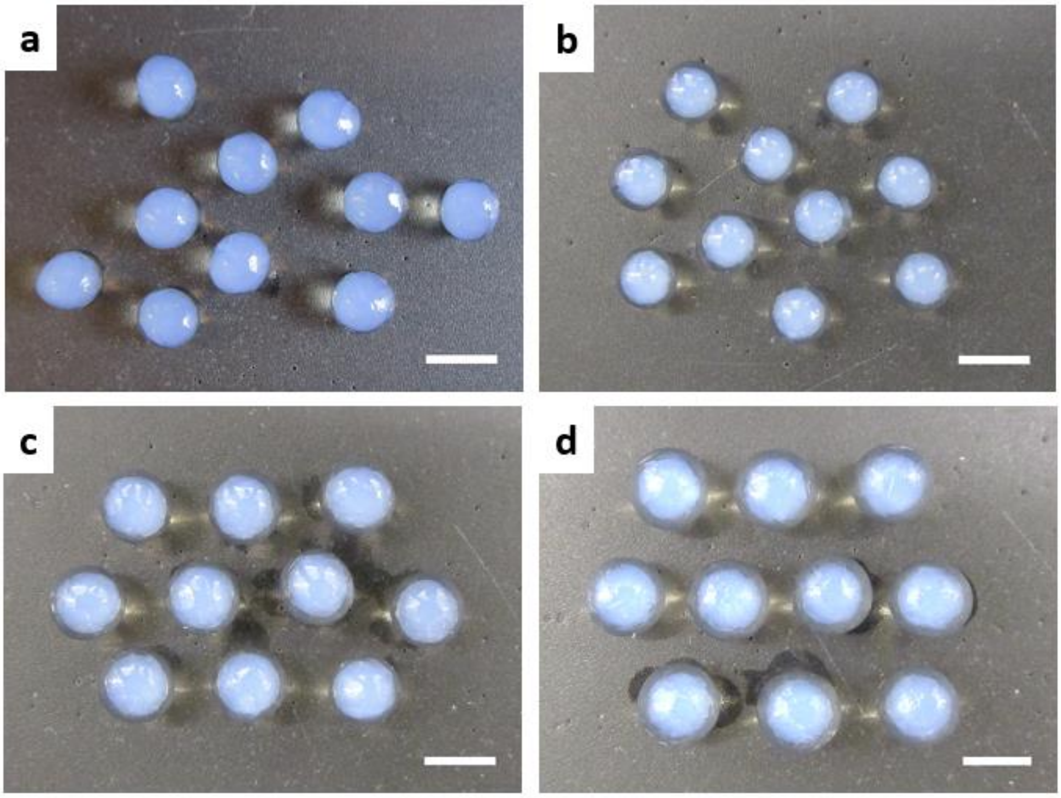

Figure S3. a-d) Optical images of capsules for different preparation conditions: a) w_{CaCl_2}= 0.02 and 18G dispensing tip, b) w_{CaCl_2}= 0.04 and 22G dispensing tip, c) w_{CaCl_2}= 0.04 and 18G dispensing tip, d) w_{CaCl_2}= 0.06 and 18G dispensing tip. Scale bars are 5 mm.

S.4. Design of Capsule Dimensions

The radius of a capsule can be estimated by the sum of the inner core radius and the shell thickness. In this work, the capsule thickness and size are easily controlled by varying the calcium concentration and the dispensing tip size, respectively. As shown in **Figure 2h**, the shell thickness is linearly correlated with the calcium concentration. Further extrapolation of this linear correlation (**Figure S4**) shows that it passes through the origin, which is reasonable because no shell can be formed without $CaCl_2$ being added. For the lower limit, the shell thickness should be further decreased by decreasing the w_{CaCl_2} until the point where the capsules are too fragile to be collected. For the upper limit, the linear correlation is extrapolated up to the point where the continuous phase is saturated with $CaCl_2$ (star symbol). The star symbol corresponds to the theoretically maximum $CaCl_2$ concentration ($w_{CaCl_2,max}$) that can be achieved in the nanoemulsion system. With the solubility of $CaCl_2$ in water (74.5 g/100 mL at 20°C)[2] and the water volume in the continuous phase (3 g sucrose solution × 75 wt.% = 2.25 g water ~ 2.25 mL water), $w_{CaCl_2,max}$ can be approximated as **Equation S2**. The shell thickness is extrapolated to be 6.44 mm for the $w_{CaCl_2,max}$.

$$w_{CaCl_2,max} = \frac{2.25\times0.745}{5} = 0.335 \text{ (g CaCl}_2\text{/g nanoemulsion)} \quad \text{(S2)}$$

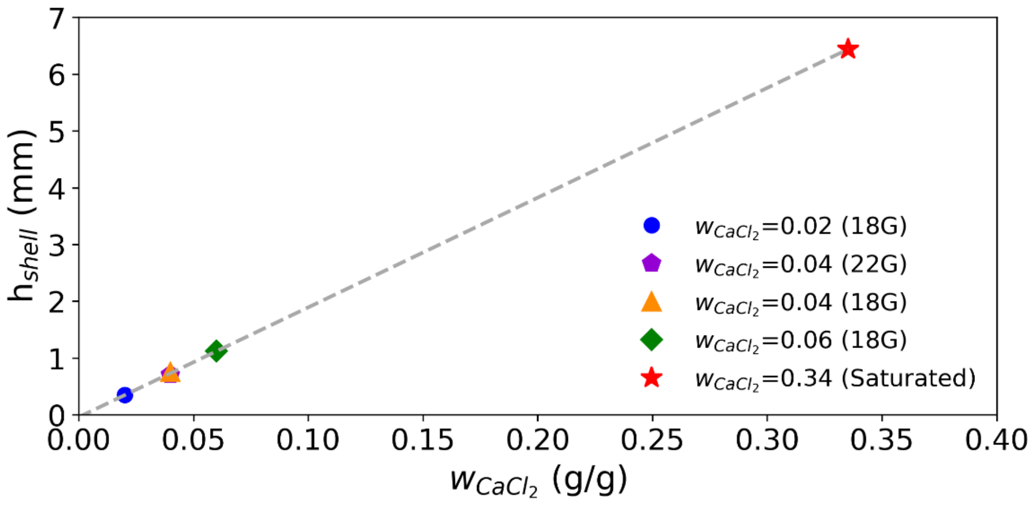

Figure S4. Linear correlation between shell thickness and w_{CaCl_2} with extrapolation to both saturated conditions and zero w_{CaCl_2}.

Since we use a dripping method to generate droplets, the inner core radius (r_{core}) can be described by Tate's law[1]:

$$r_{core} \propto \left(\frac{d_T \gamma_{NE}}{\rho_{NE} g}\right)^{\frac{1}{3}} \tag{S3}$$

where d_T is the outer diameter of the dispensing tip, γ_{NE} is the surface tension of the nanoemulsion, ρ_{NE} is the density of the nanoemulsion, g is the acceleration of gravity (9.8 m^2/s). **Figure S5** shows the linear correlation for the origin and the four data points (five points in total, R^2 = 0.99). This correlation can provide a criterion for designing the inner core radius of capsules. Alternatively, one could use centrifugal forces to produce even smaller droplets and hence inner cores. We have a previous publication which describes the centrifugal particle generation process in detail.[3]

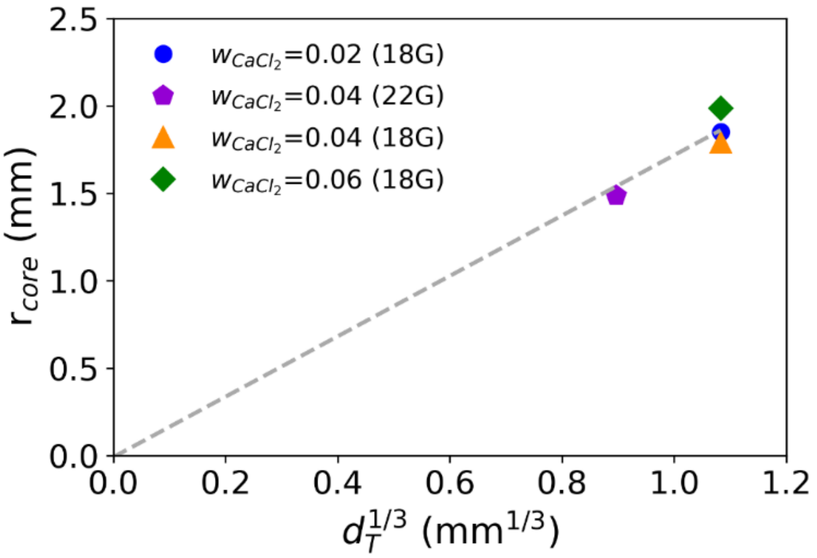

Figure S5. Linear correlation between core radius (r_{core}) and the cube root of the tip outer diameter ($d_T^{1/3}$).

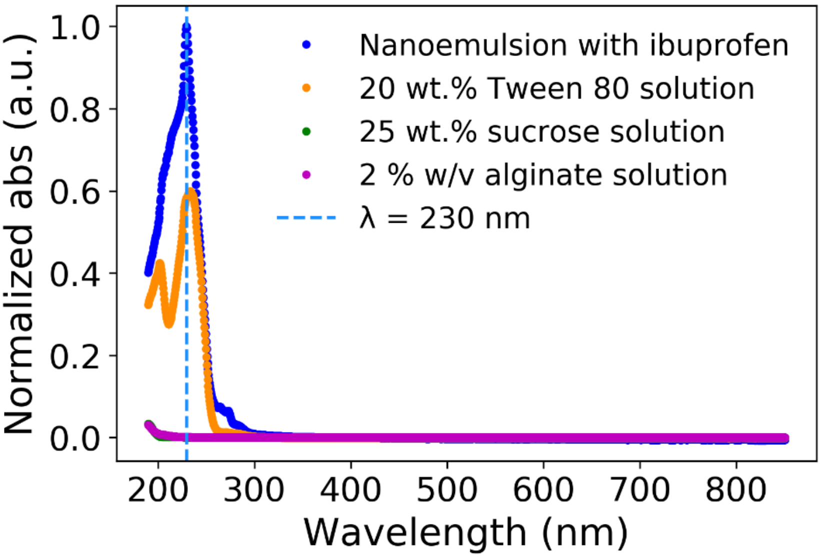

Figure S6. UV-Vis spectra for the nanoemulsion and its components.

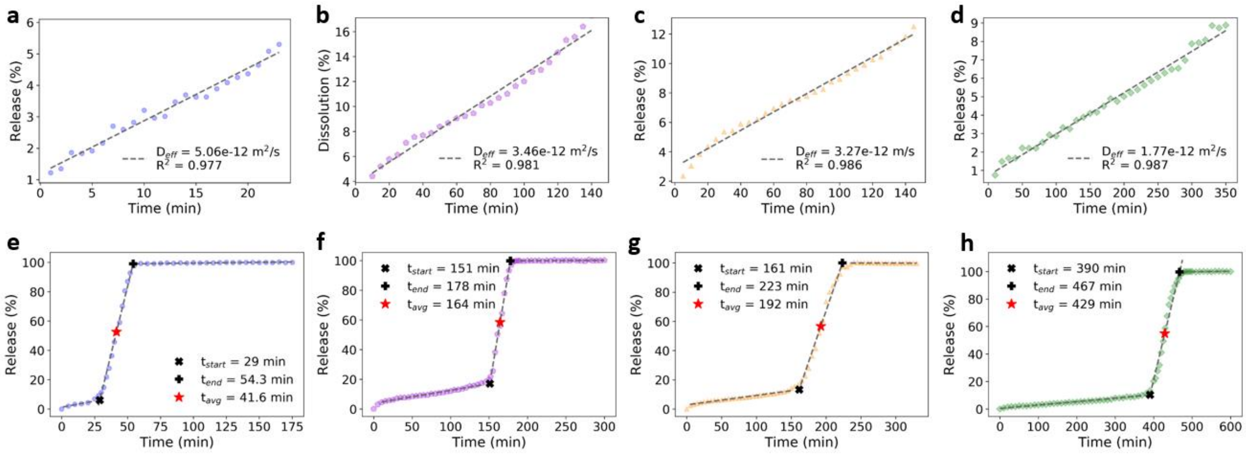

Figure S7. a-d) Determination of effective diffusivities (D_{eff}) from the slope in the early diffusion regime (*R1*): a) w_{CaCl_2}= 0.02 and 18G dispensing tip, b) w_{CaCl_2}= 0.04 and 22G dispensing tip, c) w_{CaCl_2}= 0.04 and 18G dispensing tip, d) w_{CaCl_2}= 0.06 and 18G dispensing tip. e-h) Determination of bursting time for different preparation conditions: e) w_{CaCl_2}= 0.02 and 18G dispensing tip, f) w_{CaCl_2}= 0.04 and 22G dispensing tip, g) w_{CaCl_2}= 0.04 and 18G dispensing tip, h) w_{CaCl_2}= 0.06 and 18G dispensing tip.

S.5. Fitting the Bursting Events with Cumulative Distribution Functions

The bursting events occurring in the bursting regime (*R2*) should be statistically random, and the release profile should follow the behavior of a cumulative distribution function (CDF). To fit a CDF to the bursting regime (*R2*) of the release profile, we extracted the release profile $R(t)$ from 10% to 100% (excluding the early diffusion release), and rescaled this 90% release into a CDF $\varphi(t)$ ranging from 0 to 100%. The $\varphi(t)$ represents the cumulative probability of capsule bursting. The $\varphi(t)$ is then transformed into $\varphi(\tau)$ with $\tau = t - t_{avg}$ to shift the distribution mean to $\tau = 0$. With the above setting, the only unknown parameter required to be fitted is the standard deviation σ in the following CDF $\varphi(\tau)$:

$$\varphi(\tau) = \frac{1}{2}\left[1 + \text{erf}\left(\frac{\tau}{\sigma\sqrt{2}}\right)\right] \tag{S4}$$

The results of the CDF fitting are shown in **Figure S8a-d**. When the w_{CaCl_2} or the dispensing tip size increases, a larger σ is observed. The fitted CDF can be further transformed into a probability density function $f(\tau)$ (PDF) to represent the release rate in the bursting regime. The $f(\tau)$ is transformed back to $f(t)$, which is further rescaled back to $R(t)$. **Figure S8e** shows the release rate profiles of capsules for different preparation conditions. The PDF accurately follows the bursting release rates calculated from the experimental data.

$$f(\tau) = \frac{1}{\sigma\sqrt{2\pi}} \exp\left[-\frac{\tau^2}{2\sigma^2}\right] \tag{S5}$$

$$\frac{\Delta R(t)}{\Delta t} = f\left(t - t_{avg}\right) \times 90\% \tag{S6}$$

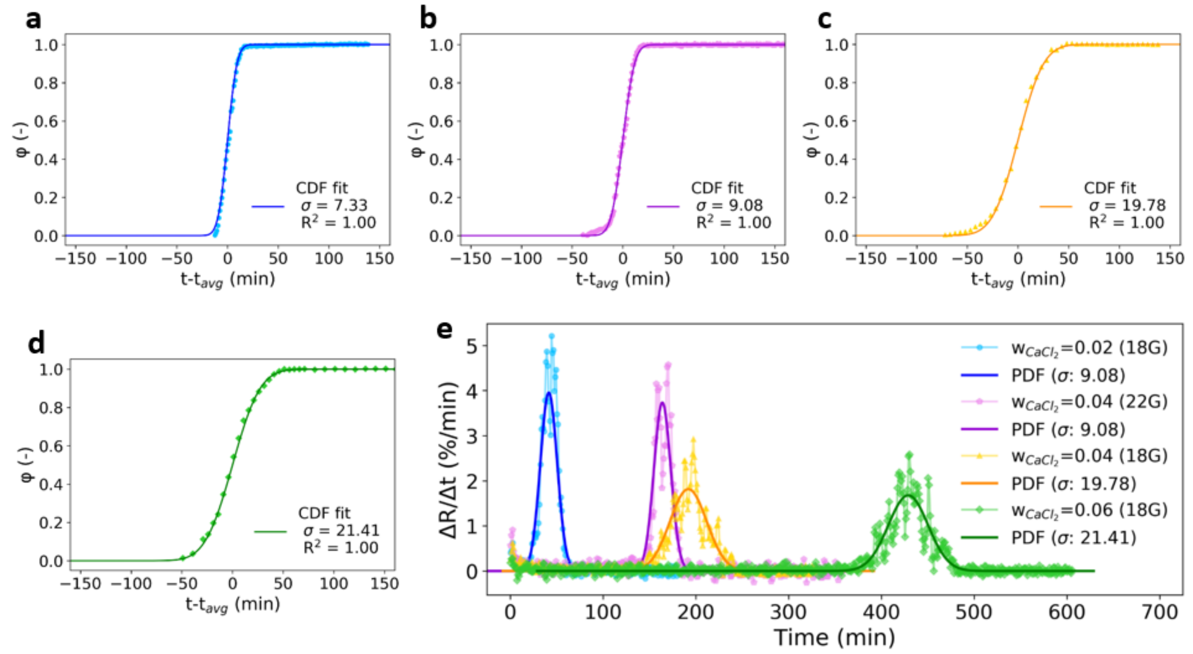

Figure S8. a-d) Fitting the bursting regime (*R2*) with a cumulative distribution function (CDF): a) w_{CaCl_2}= 0.02 and 18G dispensing tip, b) w_{CaCl_2}= 0.04 and 22G dispensing tip, c) w_{CaCl_2}= 0.04 and 18G dispensing tip, d) w_{CaCl_2}= 0.06 and 18G dispensing tip. e) Release rates ($\Delta R/\Delta t$) of capsules for different preparation conditions: the connected points are calculated from the experimental data, and the solid lines are probability density functions with the standard deviations (σ) obtained from the CDF fitting.

S.6. Preparation of Alginate Beads for Nanoemulsion Encapsulation

Nanoemulsion-loaded alginate beads were prepared by dripping alginate nanoemulsions into a calcium gelation bath (**Figure S9a**). The alginate nanoemulsions with three different alginate concentrations show droplet sizes between 50 to 55 nm (**Figure S9b**). The droplet size slightly decreases as the alginate concentration increases, because the continuous phase becomes more viscous, which provides a larger shear to create smaller droplets. The nanoemulsion-laden alginate beads are shown in **Figure S9c-e**. The bead radii are similar for different alginate concentrations (**Figure S9f**). The sphericity factors (SFs) for the three conditions are no greater than a threshold of 0.05 (**Figure S9g**). The SF slightly decreases with increasing alginate concentration because of a higher viscosity to favor the formation of more spherical alginate beads.

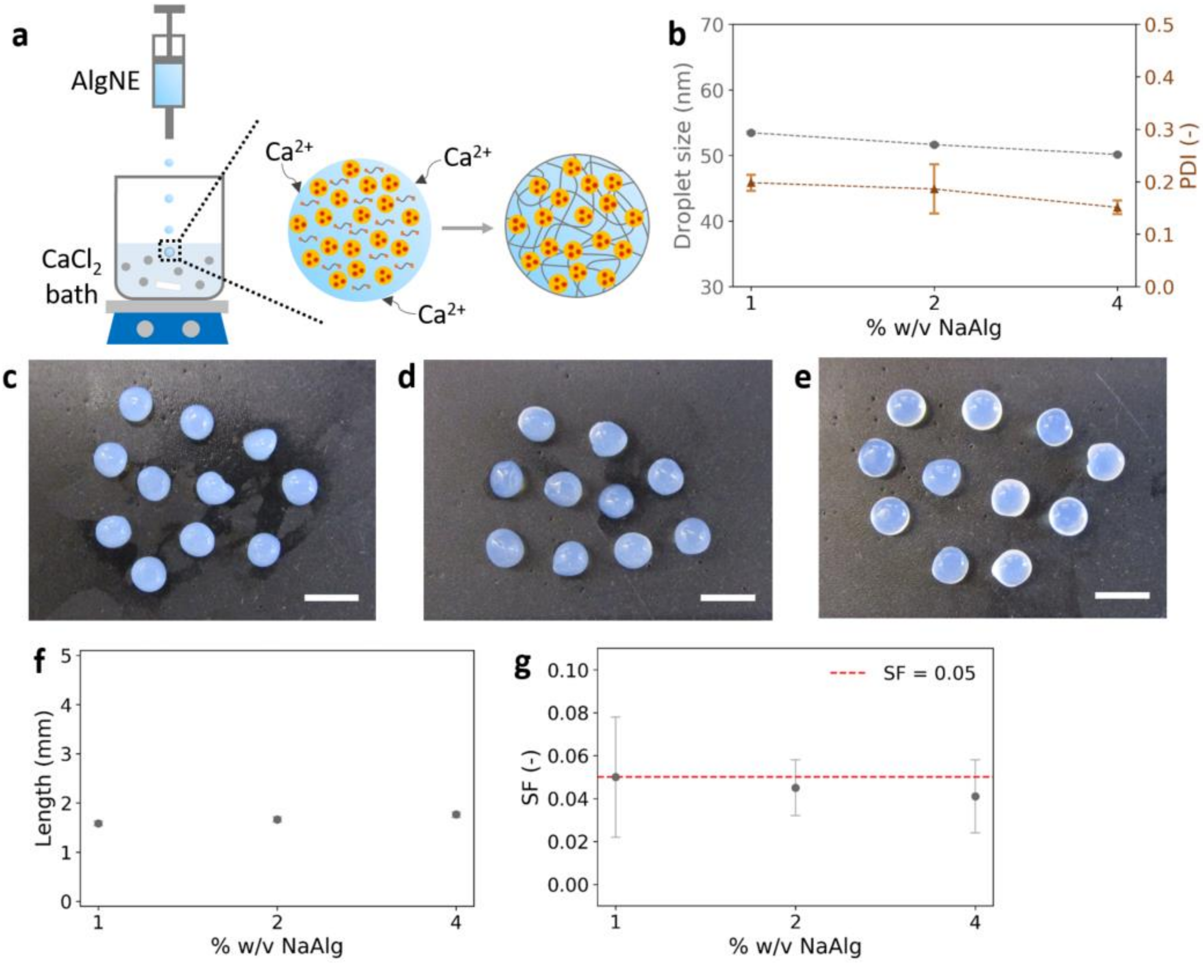

Figure S9. a) Schematic diagram of the formation of alginate beads for nanoemulsion encapsulation: the calcium ions diffuse in and ionically crosslink the alginate molecules. b) Droplet size and polydispersity index for the alginate nanoemulsions prepared with sodium alginate concentrations C_{NaAlg} of 1, 2, and 4% w/v. c-e) Optical images of beads prepared with an 18G dispensing tip and different sodium alginate concentrations: c) C_{NaAlg}= 1% w/v, d) C_{NaAlg}= 2% w/v, and e) C_{NaAlg}= 4% w/v. f) Bead radii for different preparation conditions. g) Sphericity factors (SF) of the beads for different preparation conditions. All scale bars are 5 mm.

S.7. Release Behavior of Nanoemulsion-Loaded Beads

With the linear calibration curve (**Figure 3c**), release tests are conducted to obtain the release profiles of beads for the three different alginate concentrations (**Figure S10a**). Based on the observation of the release profiles, we separate each release curve into three regimes: diffusion-controlled (*R1*), erosion-controlled (*R2*), and post-release (*R3*) regimes. During the diffusion-controlled (*R1*) regime, no significant bead erosion was observed, and the release profile has a tendency to level off over time. The transition time point (t_{trans}) from the *R1* to *R2* is defined as the point where the slope of the release $R(t)$ stops decreasing and starts to increase again. The t_{trans} is determined to be 15, 30, and 45 s for the C_{NaAlg} of 1, 2, and 4% w/v, respectively. Finally, the release process enters the *R3* as the absorbance signal reaches a saturated value indicating complete release of the cargo. **Figure S10b** shows the release rates for the three different alginate beads. For each alginate concentration, the release rate increases and develops a peak after the release mechanism transitions to the *R2* regime. In the *R2* regime, the alginate beads degrade significantly, which accelerates the nanoemulsion release. The sharpness of the peaks depends on the crosslinking density of the alginate beads. The alginate beads prepared from a lower alginate concentration possess a lower crosslinking density, which leads to a sharper peak of the release rate. **Figure S10c** shows the retention of the nanoemulsion suspension in beads for different preparation conditions. The retention rate increases as the alginate concentration increases, and the best retention is about 58.8% for the 4% w/v alginate beads. To further analyze the release profiles, **Equation S7** and **Equation S8** based on Peppas power law are used to fit the *R1* and *R2* regimes, respectively.[4]

$$R(\%) = kt^n \qquad \text{(S7)}$$

$$R(\%) = k(t - t_{trans})^n + R_{trans}(\%) \qquad \text{(S8)}$$

where k is a geometric constant for a hydrogel system, and n is the diffusional exponent representing the release mechanism, $R_{trans}(\%)$ is the $R(\%)$ at t_{trans}. For spherical matrices,

the values of n = 0.43, 0.43 < n < 0.85, n = 0.85, and n > 0.85 represent Fickian diffusion, anomalous (non-Fickian) transport, Case II transport, and Super Case II transport, respectively.[4] The results of the power law fitting are shown in **Figure S10d-f.** The exponent n values are 0.43 < n < 0.85 and n > 0.85 for the *R1* and *R2* regimes, respectively. This indicates that the *R1* and *R2* regimes belong to anomalous (non-Fickian) transport and Super Case II transport, respectively. In the *R1* regime, the release is mainly diffusion-controlled with some anomalous behavior due to relaxation and erosion. In contrast, the release is mainly erosion-controlled in the *R2* regime.[5]

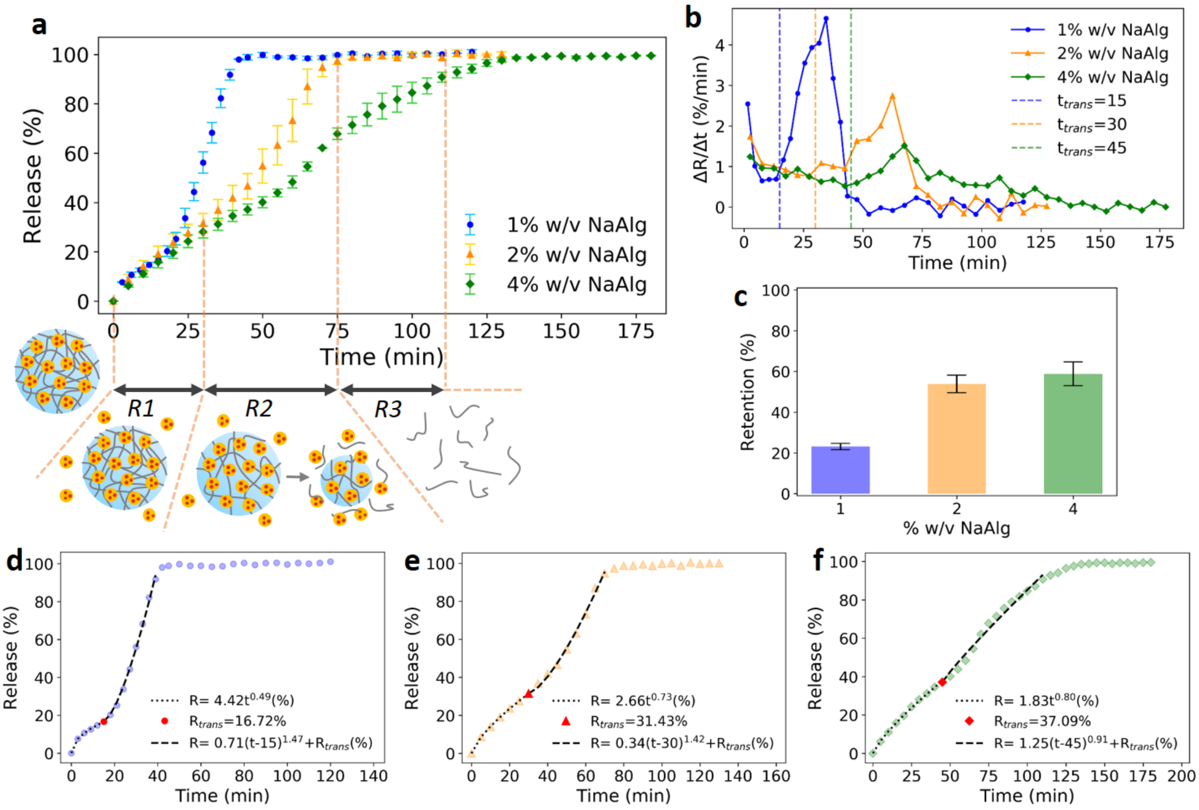

Figure S10. a) Release profiles using a USP Dissolution Apparatus II of beads for different preparation conditions. The schematic images below the release profiles depict the release mechanisms for different regimes corresponding to the C_{NaAlg}= 2% w/v (which also apply to other curves). *R1*, *R2*, and *R3* are diffusion-controlled, erosion-controlled, and post-release regimes. b) Release rates ($\Delta R/\Delta t$) of beads for different preparation conditions. c) Retention of the nanoemulsion suspension in beads for different preparation conditions. d-f) Fitting the release profiles with time-dependent power law functions: d) C_{NaAlg}= 1% w/v, e) C_{NaAlg}= 2% w/v, and f) C_{NaAlg}= 4% w/v.

S.8. Tables for Retention Calculation

Table S2. Retention calculation for alginate capsules.

w_{CaCl_2}(tip size)	0.02 (18G)	0.04 (22G)	0.04 (18G)	0.06 (18G)
n_c	40	70	40	40
m_d (mg)	13.0±0.2	7.0±0.1	12.8±0.3	12.7±0.2
m_{opt} (mg)	518.6±8.8	488.3±5.6	513.1±11.1	506.6±9.8
I_{sat}	0.484±0.007	0.522±0.003	0.508±0.006	0.516±0.004
m_{act} (mg)	443.3±6.4	465.4±5.6	477.5±2.7	472.2±3.4
R_t (%)	85.5±1.9	95.3±1.6	93.1±2.1	93.2±1.9

Table S3. Retention calculation for alginate beads.

C_{NaAlg} (% w/v)	1	2	4
n_c	40	40	40
m_d (mg)	12.6±0.5	12.6±0.4	12.7±0.3
m_{opt} (mg)	502.4±18.8	502.9±14.5	506.9±14.0
I_{sat}	0.127±0.007	0.296±0.022	0.326±0.031
m_{act} (mg)	116.3±6.7	270.5±20.1	298.1±28.5
R_t (%)	23.1±1.6	53.8±4.3	58.8±5.8

References

[1] E. S. Chan, B. B. Lee, P. Ravindra, D. Poncelet, *J. Colloid Interface Sci.* **2009**, *338*, 63.

[2] D. R. Lide, *CRC Handbook of Chemistry and Physics*, CRC Press, **2004**.

[3] H. B. Eral, E. R. Safai, B. Keshavarz, J. J. Kim, J. Lee, P. S. Doyle, *Langmuir* **2016**, *32*, 7198.

[4] P. L. Ritger, N. A. Peppas, *J. Control. Release* **1987**, *5*, 37.

[5] S. Maity, B. Sa, *Int. J. Biol. Macromol.* **2014**, *68*, 78.
